# Changes in lung volume estimated by electrical impedance tomography during apnea and high-flow nasal oxygenation: A single-center randomized controlled trial

**DOI:** 10.1371/journal.pone.0273120

**Published:** 2022-09-28

**Authors:** Thomas Riedel, Fabian Bürgi, Robert Greif, Heiko Kaiser, Thomas Riva, Lorenz Theiler, Sabine Nabecker

**Affiliations:** 1 Division of Paediatric Intensive Care Medicine, Department of Paediatrics, University Children’s Hospital, University of Bern, Bern, Switzerland; 2 Department of Anaesthesiology and Pain Medicine, Bern University Hospital, University of Bern, Bern, Switzerland; 3 School of Medicine, Sigmund Freud University Vienna, Vienna, Austria; 4 Centre for Anaesthesiology and Intensive Care Medicine, Hirslanden Klinik Aarau, Hirslanden Group, Aarau, Switzerland; 5 Unit for Research & Innovation in Anaesthesia, Department of Paediatric Anaesthesia, Istituto Giannina Gaslini, Genoa, Italy; 6 Department of Anaesthesia, Kantonsspital Aarau, Aarau, Switzerland; 7 Department of Anesthesiology and Pain Management, Sinai Health System, University of Toronto, Toronto, Canada; Charité - Universitätsmedizin Berlin, GERMANY

## Abstract

**Background:**

Previous studies concerning humidified, heated high-flow nasal oxygen delivered in spontaneously breathing patients postulated an increase in functional residual capacity as one of its physiological effects. It is unclear wheter this is also true for patients under general anesthesia.

**Methodology:**

The sincle-center noninferiority trial was registered at ClinicalTrials.gov (NCT NCT03478774). This secondary outcome analysis shows estimated differences in lung volume changes using electrical impedance tomography between different flow rates of 100% oxygen in apneic, anesthetized and paralyzed adults prior to intubation. One hundred and twenty five patients were randomized to five groups with different flow rates of 100% oxygen: i) minimal-flow: 0.25 l.min^-1^ via endotracheal tube; ii) low-flow: 2 l.min^-1^ + continuous jaw thrust; iii) medium-flow: 10 l.min^-1^ + continuous jaw thrust; iv) high-flow: 70l.min^-1^ + continuous jaw thrust; and v) control: 70 l.min^-1^ + continuous video-laryngoscopy. After standardized anesthesia induction with non-depolarizing neuromuscular blockade, the 15-minute apnea period and oxygen delivery was started according to the randomized flow rate. Continuous electrical impedance tomography measurements were performed during the 15-minute apnea period. Total change in lung impedance (an estimate of changes in lung volume) over the 15-minute apnea period and times to 25%, 50% and 75% of total impedance change were calculated.

**Results:**

One hundred and twenty five patients completed the original study. Six patients did not complete the 15-minute apnea period. Due to maloperation, malfunction and artefacts additional 54 measurements had to be excluded, resulting in 65 patients included into this secondary outcome analysis. We found no differences between groups with respect to decrease in lung impedance or curve progression over the observation period.

**Conclusions:**

Different flow rates of humidified 100% oxygen during apnea result in comparable decreases in lung volumes. The demonstrated increase in functional residual capacity during spontaneous breathing with high-flow nasal oxygenation could not be replicated during apnea under general anesthesia with neuromuscular blockade.

## Introduction

High-flow nasal oxygen (HFNO) is the administration of humidified, heated, and blended air/oxygen via nasal cannulas at rates of up to 2 l.kg^-1^ min^-1^ or a maximum of 80 l.kg^-1^ min^-1^. It was first described for the relief of sleep-related oropharyngeal airway obstruction [1]. It was used later in neonatal intensive care units for treatment of apnea in premature infants [2]. Since then, it is used widely in pediatric intensive care medicine mainly for treatment of respiratory distress [3], for respiratory support after extubation [4] in premature children, and for treatment of bronchiolitis in infants [5].

In adult intensive care medicine it is used to improve oxygenation in hypoxemic respiratory failure because it is easy to use and requires minimal cooperation from patients [6]. In anesthesia it was first described in 2015 in a case series of 25 patients [7]. HFNO prolonged apnea time, and was also suspected to improve ventilation during apnea. It was therefore named transnasal humidified rapid-insufflation ventilatory exchange (THRIVE). HFNO gained popularity for both adults and children for tubeless laryngeal surgery [7–9] and for prolongation of apnea time during airway management for patients with potentially difficult airways [7, 10]. Proposed physiological mechanisms of increasing apnea time during HFNO include positive airway pressure generation and an increase in functional residual capacity [11–13].

Multiple studies investigated the effects of high-flow nasal oxygen during respiratory distress [14, 15]. Spontaneously breathing patients using HFNO seem to benefit from decreased inspiratory effort, improved lung volume and lung compliance [16]. Positive airway pressure generation was absent in apneic patients with an open mouth, and minimal with a closed mouth [17].

Two previous studies suggested active carbon dioxide washout during apnea because apnea time was prolonged during HNFO and the rate of carbon dioxide was lower when compared to historical data [7, 8]. However, two randomized controlled trials using different oxygen flow rates in apneic pediatric patients detected no differences in carbon dioxide rise [10, 18]. In a single center randomized controlled trial in paralyzed adults, our group found no differences in carbon dioxide rise at different flow rates between 0.25 and 70 l.min^-1^ [19]. Therefore, the postulated carbon dioxide washout could not be verified during apnea.

The present study is a secondary outcome analysis of this previously published randomized controlled trial [19, 20]. We aimed to assess the dynamics of lung impedance using electrical impedance tomography (EIT) in apneic patients, to determine whether different oxygen flow rates during apnea may cause less atelectasis formation and/or an increase in functional residual capacity.

To achieve this we estimated the changes in lung volume by EIT during HFNO at different flow rates in apneic, anesthetized and paralyzed adult elective surgical patients prior to intubation.

## Methods

The Cantonal Ethics Committee of Bern approved the study (2018–00293). It was registered at ClinicalTrials.gov (NCT NCT03478774, Primary Investigator: Lorenz Theiler. Registration date March 27, 2018. Written informed consent was obtained by the research team from all participants. This single-center randomized controlled trial was performed in the Department of Anaesthesiology and Pain Medicine, at the Bern University Hospital in Bern, Switzerland between March 2018 and December 2019. The primary outcome and the protocol of this project, including detailed methodology, inclusion and exclusion criteria, were published [19, 20].

Eligible study participants were adult patients aged 18 to 80 years with an ASA physical status of 1–3 who were able to speak German or French, and were scheduled for elective surgery under general anesthesia. After screening for inclusion and exclusion criteria, patients were recruited during the standard preadmission clinic.

### Study procedure

The detailed methods are published elsewhere [18, 20]. In brief, patients were monitored according to the local standard of anesthesia care with additional placement of two transcutaneous sensors for carbon dioxide and oxygen measurements (TCM 4 and TCM 5; both Radiometer, Krefeld, Germany), and a thoracic EIT belt (PulmoVista 500; Dräger, Lübeck, Germany). A lose-fitting belt with 16 evenly spaced electrodes was placed around the chest of each patient between the 4^th^ and 6^th^ intercostal space, in a thoracic median plane.

After standard pre-oxygenation, anesthesia was induced using a target-controlled infusion of propofol and remifentanil. Neuromuscular blockade was induced using Rocuronium; adequacy was verified with a train-of-four value of 0 before onset of apnea, every 5 minutes throughout the procedure, and by visual absence of even small diaphragmatic movements on the EIT screen. The patient’s position remained unchanged during the study period.

After verification of successful facemask ventilation, patients were randomized to one of five study groups using a computer-generated sequence kept in sealed envelopes. The randomization list was generated by a study nurse of the Department of Anaesthesiology and Pain Medicine of the Bern University Hospital, who was not a member of the study group. Then the apneic period was started. The five studied groups were:

- Minimal-flow group: 0.25 l.min^-1^ oxygen via endotracheal tube
- Low-flow group: 2 l.min^-1^ oxygen + continuous jaw thrust
- Medium-flow group: 10 l.min^-1^ oxygen + continuous jaw thrust
- High-flow group: 70 l.min^-1^ oxygen + continuous jaw thrust
- Control group: 70 l.min^-1^ oxygen + continuous laryngoscopy with a McGrath MAC video laryngoscope (Medtronic, Dublin, Ireland).

Blinding of study personnel was not feasible, but patients were blinded to their group allocation. All patients received 100% oxygen. We delivered high-flow humidified oxygen via a high-flow nasal cannula (Optiflow™ MR 850 system Fisher&Paykel™, Auckland, New Zealand). Medium and low-flow humidified oxygen was delivered using Aquapak Hudson RCI (Teleflex, Wayne, Pennsylvania, USA) and a flow-meter (Carbamed digi-flow, Switzerland) using a standard nasal cannula (O_2_-Star nasal cannula curved, Dräger, Lübeck, Germany). Minimal-flow oxygen was delivered via a standard tracheal tube, using the circuit of a Dräger Primus anesthesia machine (Dräger, Lübeck, Germany) to ensure the delivery of exactly 0.25 l.min^-1^.

We confirmed upper airway patency visually using a nasopharyngeal fiberscope (EF-N slim, Acutronic, Hirzel, Switzerland) immediately after the start of apnea, and at 7 and 14 min of apnea. If the airway was not patent, an oropharyngeal tube (Guedel airway, Intersurgical, Workingham, Berkshire, UK) was inserted [21]. If the airway was then still not patent, the study intervention was terminated.

There were several pre-defined study termination criteria: SpO_2_ <92%, transcutaneous CO_2_ >100 mmHg, pH <7.1, potassium >6 mmol l^-1^, and apneic period reaching 15 min. If any of these occured, immediate bag-mask ventilation was started. The attending anesthesiologists then performed airway management at their discretion. After successful intubation, the anesthesiologist performed a standardized manual airway recruitment maneuver (sustained manual inflation to an airway pressure of 40 mbar for 15 seconds) [22]. The patient was then connected to the circuit of a Dräger Primus anesthesia machine (Dräger, Lübeck, Germany) and mechanical ventilation was commenced with tidal volumes of 6 ml.kg^-1^ lean body weight.

### Measurements

Thoracic EIT measurements were continuously recorded at a frame rate of 30 Hz during the whole study period. We excluded patients with incomplete measurements due to malfunction of the EIT device, if total malfunction of the device occurred, if there were major artefacts in the EIT signal, and if any of the predefined study termination criteria (especially SpO_2_ <92% before end of the 15 minutes apnea time) were reached. The EIT images were reconstructed from the raw data based on the Graz consensus reconstruction algorithm for electrical impedance tomography (GREIT) using the torso mesh function [23, 24].

**Fig 1:**
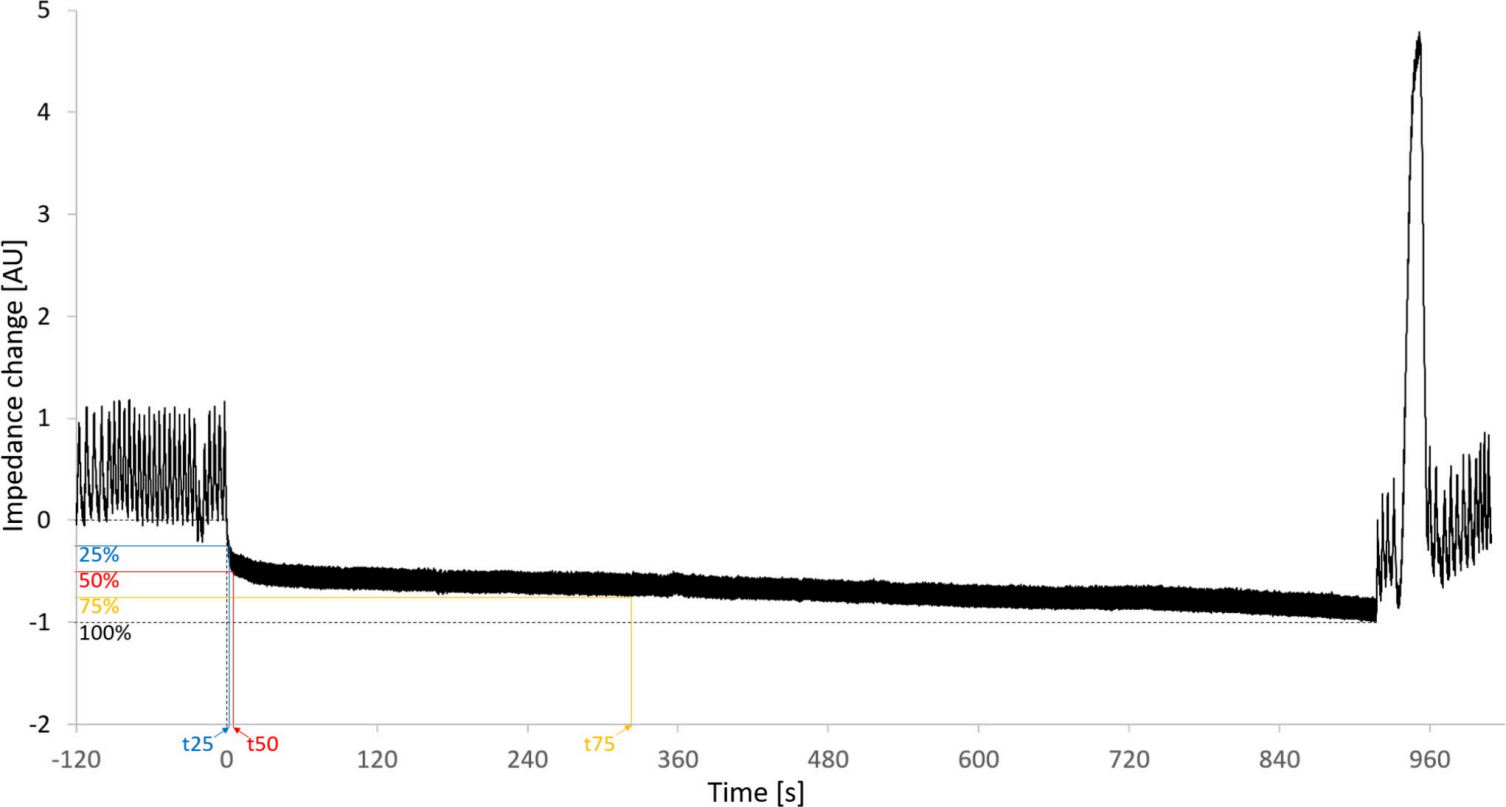
Example of a typical impedance signal time course. t25, t50 and t75 define the time to 25%, 50% and 75% respectively of total impedance reduction from baseline.

The change in lung volume was estimated by calculating lung impedance change, normalized to the impedance amplitude during mechanical ventilation at 6 ml.kg^-1^ using adapted customized code (Matlab R2021a; The MathWorks Inc., Nattick, MA, USA) [25, 26]. Additionally, to detect potential differences in curve progression of the change in lung impedance, the time to 25%, 50% and 75% of total impedance reduction from baseline was calculated (Fig 1). All analyses were performed for the global impedance signal as well as for 4 regions of interest (anterior, mid-anterior, mid-posterior, and posterior).

### Statistical analysis

The necessary sample size was calculated for the main outcome of the original study (difference in carbon dioxide increase). It was 22 patients per group; 25 patients were recruited per group [19]. No additional sample size calculation was performed for this secondary outcome analysis.

Data are reported as median [IQR] or mean ± SD. A probability of less than 0.05 was considered significant. Differences between groups were analyzed using Kruskal-Wallis test, corrected for multiple comparisons. All statistical analyses were performed with StatsDirect 3.3.5 (StatsDirect Ltd, Wirral, UK).

## Results

**Fig 2:**
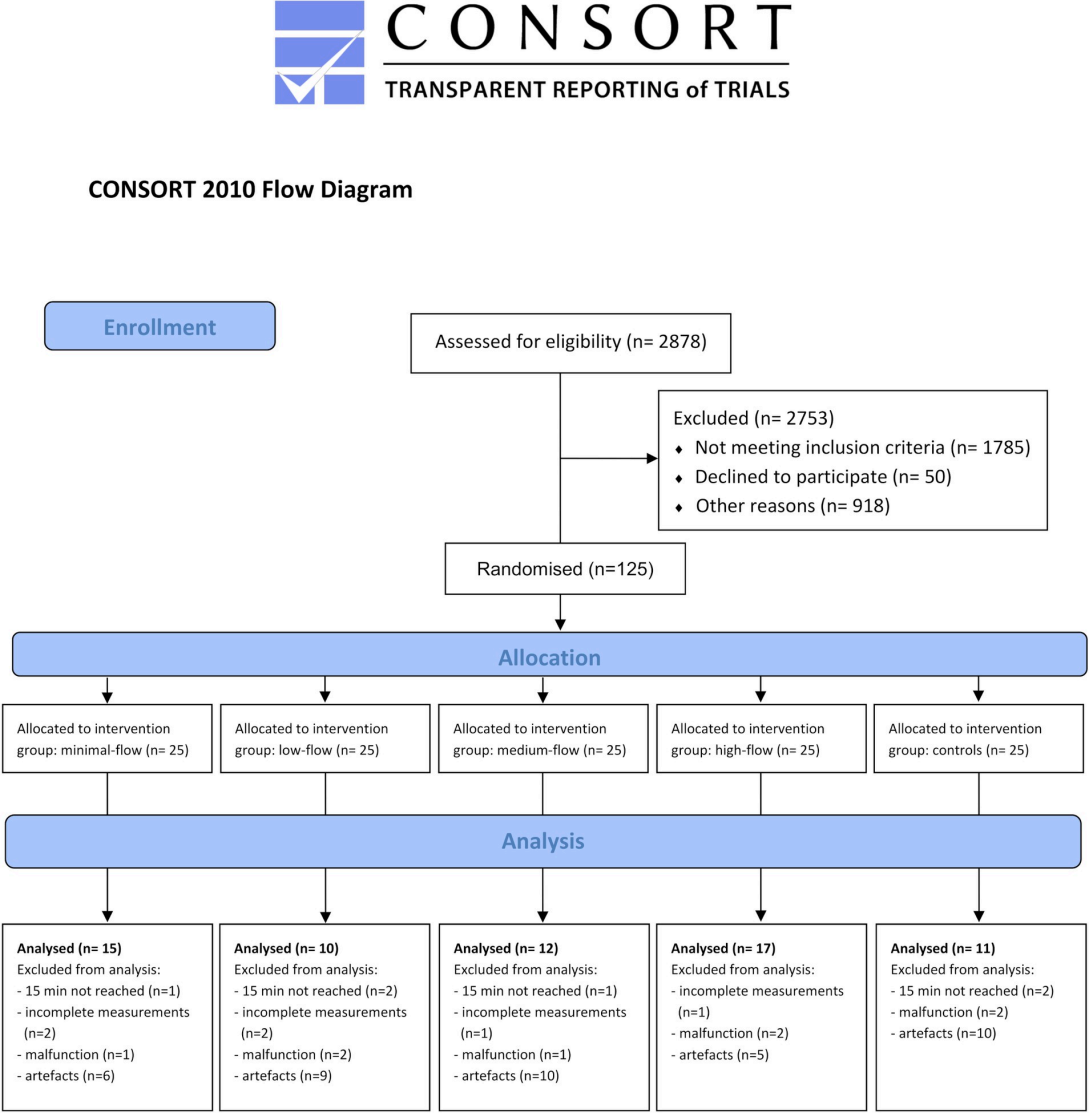
Consolidated standards of reporting trials flow diagram.

From the 125 participants of the original study, 65 could be included in the present analysis (six patients were excluded because of malfunction of the measurement device resulting in incomplete measurements; eight patients were excluded due to total malfunction of the device, 40 patients had to be excluded because of major artefacts in the EIT signal (sudden, unexplained steps in impedance; movement artefacts), and 6 patients reached the predefined study termination criterion of SpO_2_ below 92% before the end of the 15-minute apnea period). The number of patients per randomized group included in this analysis was: (i) minimal-flow: 15 patients; (ii) low-flow: 10 patients; (iii) medium-flow: 12 patients; (iv) high-flow: 17 patients; (v) control: 11 patients (Fig 2). For baseline characteristics see Table 1.

**Table 1:** 

	minimal-flow	low-flow	medium-flow	high-flow	controls
	n = 15	n = 10	n = 12	n = 17	n = 11
Female	6 (40)	5 (50)	7 (58)	7 (41)	6 (55)
Age y	44 ± 17	46 ± 15	47 ± 18	49 ± 20	48 ± 20
Weight kg	76 ± 15	75 ± 17	73 ± 17	77 ± 19	69 ± 14
Height cm	172 ± 9	171 ± 8	172 ± 10	170 ± 13	169 ± 11
Body mass index kg.m^-2^	25 ± 3.7	24 ± 3.5	23 ± 3.5	25 ± 4.2	22 ± 3.9
Smoker/ex-smoker	5 (33)	5 (50)	3 (25)	4 (24)	5 (45)
ASA physical status					
I	7 (47)	2 (20)	2 (17)	4 (24)	3 (27)
II	7 (47)	7 (70)	10 (83)	12 (70)	7 (64)
III	1 (4)	1 (10)	0 (0)	1 (6)	1 (9)

Values given as number (percentage) or mean ± standard deviation; ASA American Society of Anesthesiologists

**Fig 3:**
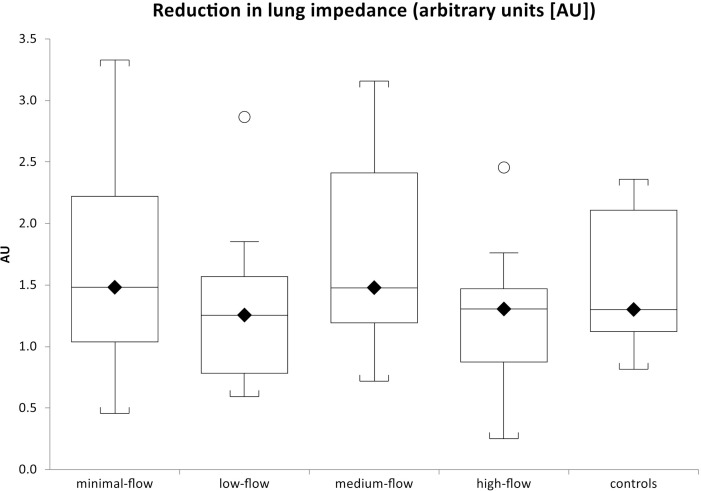
Total reduction in lung impedance normalized for impedance amplitude at tidal volumes of 6ml.kg^-1^ lean body weight at different flow rates. No significant differences between groups.

The reduction in lung impedance normalized to the amplitude during mechanical ventilation at tidal volumes of 6ml.kg^-1^ lean body weight for all patients was 1.45 ± 0.72. Fig 3 shows the lung impedance change for each group, without significant differences between them, neither for the whole lung (p = 0.34) nor for the different regions of interest: p = 0.94 (anterior), p = 0.41 (mid-anterior), p = 0.48 (mid-posterior), and p = 0.27 (posterior). Time to 25%, 50% and 75% of total impedance change from baseline also did not differ significantly between groups (Table 2). We could not detect an impact of the tracheal tube on lung impedance in the minimal-flow group.

**Table 2:** 

	minimal- flow	low- flow	medium- flow	high- flow	controls	
**Time to 25%**	1.3s [1.0–1.4]	1.4s [0.7–1.8]	1.2s [1.0–1.9]	1.5s [0.9–1.8]	1.3s [1.0–1.7]	p = 0.99
**Time to 50%**	5.1s [3.6–8.5]	6.2s [3.1–18.7]	5.7s [2.7–6.4]	4.9s [3.1–11.8]	5.1s [2.9–7.2]	p = 0.97
**Time to 75%**	56.1s [39.5–101.6]	59.6s [38.0–420.4]	54.7s [27.1–61.4]	52.8s [16.4–92.5]	49.2s [31.6–55.0]	p = 0.77

median [IQR] time to 25%, 50% and 75% of total impedance change from baseline; p-values derived from Kruskal-Wallis test

## Discussion

This secondary outcome analysis of a previously published single-center randomized controlled non-inferiority trial on different flow rates of high-flow nasal oxygenation, demonstrates that the decrease in lung volume during apnea measured with electrical impedance tomography is independent of flow rates between 0.25 l.min^-1^ and 70 l.min^-1^.

HFNO was first applied in spontaneously breathing preterm infants and pediatric patients with respiratory distress (mainly caused by viral bronchiolitis) [27, 28]. Later, studies showed beneficial outcomes in adult patients with acute lung injury and post-extubation [29, 30]. Different physiological mechanisms have been postulated to be responsible for the clinical benefits of HFNO including reduction in work of breathing, carbon dioxide clearance, humidification of the air/oxygen mixture and increased lung volumes [31]. Researchers demonstrated an increase in end-expiratory lung impedance, which is a surrogate parameter for lung volume, that was independent of body position in healthy adults [32]. Others found similar lung recruitment with HFNO compared to non-invasive ventilation in a crossover study on adult patients with acute respiratory failure [33]. A different research group showed continuous lung recruitment with increasing flow rates up to 60 l.min^-1^ in high risk patients after extubation [34]. Our data shows that while in spontaneously breathing patients the increase of flow rates of HFNO generates an increase in functional residual capacity, the same phenomenon seems not to occur in apneic, anaesthetized and muscle relaxed patients. Lung volume loss as a surrogate parameter for atelectasis formation occured independently of flow rates and was similar in all groups studied in this analysis.

Multiple studies showed that HFNO prolongs safe apnea time and improves apneic oxygenation during intubation and airway management in adult and pediatric patients under general anesthesia [9, 10, 35–38]. However, none of these studies investigated changes in lung volume or compared different flow rates. A recent study investigated airway pressures during apneic HFNO. The researchers did not find an increase in airway pressure when the mouth of the patients remained open [17]. Our study adds further evidence that prolongation of the apnea period with HFNO occurs by an “aventilatory mass flow” of oxygen independently of the generated airway pressures or lung volumes as already described over 60 years ago [39]. Despite the evident usefulness of HFNO to prolong the safe apnea period, it is now clear that the insignificant pressures generated in the airway cannot prevent atelectasis formation, which could be even provoked by the use of a high oxygen concentrations [22, 40, 41].

Our study shows additionally that the decrease in lung volume is very rapid after the onset of apnea. Within less than 10 seconds half of the total reduction in lung volume occured in most patients. Thereafter the decline slowed down, with 25% of volume reduction after approximately 40 seconds. This fast decrease in lung volume is most likely caused by the loss of functional residual capacity approaching residual volume. Later we expect atelectasis formation during ongoing apnea. These results have two major consequences. First, no matter how fast the airway was secured in apneic patients, a substantial reduction in lung volume has already occurred. Second, the amount of lung volume loss seems to justify the performance of a recruitment maneuver after airway instrumentation [42, 43]. No patient in our study desaturated within five minutes of apnea with supplemental 100% oxygen independently of flow rates. This supports the recommendation to use any form of supplemental 100% oxygen administration during airway management [44, 45]. However, providers need to take the extensive exclusion criteria list of this study into account [19, 20].

One limitation of this secondary analysis is the high number of measurements that had to be excluded due to technical reasons. EIT is a technique developed to measure tidal ventilation. During apnea the low signal-to-noise ratio is a disadvantage because minor disturbances often lead to relevant artefacts [46, 47]. Other limitations are the single-center study design and the extensive exclusion criteria list. Those limitations should be considered in judging the generalizability of our results.

In conclusion, this study provides first insights into the decrease in lung volume in anesthetized and paralyzed adults during apneic oxygenation with 100% oxygen at flow rates between 0.25 l.min^-1^ and 70 l.min^-1^. It was comparable between all groups. The reduction in lung volume happens rapidly after the onset of apnea, which justifies routine lung recruitment maneuvers after airway instrumentation.

## Supporting information

S1 Checklist CONSORT 2010 checklist of information to include when reporting a randomised trial*. (DOCX)

S1 File (PDF)
